# *Echinacea* as a Potential Force against Coronavirus Infections? A Mini-Review of Randomized Controlled Trials in Adults and Children

**DOI:** 10.3390/microorganisms10020211

**Published:** 2022-01-19

**Authors:** Simon Nicolussi, Karin Ardjomand-Woelkart, Rainer Stange, Giuseppe Gancitano, Peter Klein, Mercedes Ogal

**Affiliations:** 1 iC-Cure Scientific, 9404 Rorschacherberg, Switzerland; 2 Department of Pharmacognosy, Institute of Pharmaceutical Sciences, University of Graz, 8010 Graz, Austria; ka.woelkart@uni-graz.at; 3 Institute of Social Medicine, Epidemiology and Health Economics, Charité—Universitätsmedizin Berlin, Corporate Member of Freie Universität and Humboldt-Universität zu Berlin, 10117 Berlin, Germany; rainer.stange@immanuelalbertinen.de; 4 1st “Tuscania” Paratrooper Regiment Carabinieri, Italian Ministry of Defence, 57127 Livorno, Italy; giuseppe.gancitano208@gmail.com; 5 d.s.h. Statistical Services GmbH, 85296 Rohrbach, Germany; peter.klein@dsh-statistik.de; 6 Pediatric Clinic, 6440 Brunnen, Switzerland; mercedes@ogal.ch

**Keywords:** antiviral, coronavirus, viral Load, membranous viruses, *Echinacea*, prevention, respiratory tract infections, review

## Abstract

*Echinacea purpurea* has been shown to broadly inhibit coronaviruses and SARS-CoV-2 in vitro. This review discusses the available clinical evidence from randomized, blinded and controlled human studies. Two RCTs capturing incidence of viral respiratory tract infections during *Echinacea* preventative treatment were identified including coronavirus infections. Incidence and/or viral loads were measured by RT-PCR and symptom severity was recorded. In a first study, Jawad et al. collected nasopharyngeal swabs from adults (N = 755) over 4 months of continuous prevention. Overall, 24 and 47 enveloped virus infections occurred, including 21 and 33 coronavirus detections (229E; HKU1; OC43) with Echinaforce^®^ extract (2400 mg daily) and placebo, respectively (*p* = 0.0114). In a separate study, Ogal et al. administered the same extract (1200 mg) or control for 4 months to children (4–12 years) (N = 203). *Echinacea* reduced the incidence of enveloped virus infections from 47 to 29 (*p* = 0.0038) whereas 11 and 13 coronavirus detections (229E, OC43, NL63) were counted (*p* > 0.05). Respiratory symptoms during coronavirus infections were significantly lower with area-under-curve AUC = 75.8 (+/−50.24) versus 27.1 (+/−21.27) score points (*p* = 0.0036). Importantly, viral loads in nasal secretions were significantly reduced by 98.5% in the *Echinacea* group, with Ct-values 31.1 [95% CI 26.3; 35.9] versus 25.0 [95% CI 20.5; 29.5] in the control group (*p* = 0.0479). Results from clinical studies confirm the antiviral activity found for *Echinacea* in vitro, embracing enveloped respiratory pathogens and therefore coronaviruses as well. Substantiating results from a new, completed study seem to extrapolate these effects to the prevention of SARS-CoV-2 infections. As hypothesized, the established broad antiviral activity of *Echinacea* extract appears to be inclusive for SARS-CoV-2.

## 1. Introduction

Zoonosis describes the transmission of pathogens from animals to humans. Earlier historical events of zoonosis include the transmission of *Yersinia pestis* from rodent flea (plague) or Ebola spilling over from apes to humans, causing millions of deaths in Europe and Africa. Respiratory viruses like influenza have been repeatedly seen crossing species barriers from animals to humans, triggering deadly disease outbreaks like the Spanish (H1N1), Asian (H2N2) or Hong Kong (H3N2) flu epidemics, as well as the more recent bird flu caused by avian H5N1 virus [1].

The 21st century has seen another zoonotic thread, coronaviruses, with successfully contained outbreaks of Severe Acute Respiratory Syndrome coronavirus (SARS-CoV) in 2002/2003 and Middle East Respiratory Syndrome (MERS-CoV) occurring from 2012 to 2015 [2]. Most coronaviruses can be traced back to being of animal origin, where camels, bats and civet cats are likely natural hosts or vectors [3,4]. Due to their high pathogenicity and rather short incubation period, previous coronavirus outbreaks have been relatively easy to curb, and have thus been limited to restricted areas. In contrast, SARS-CoV-2 is much trickier to contain due to its ability to disseminate through “silent transmitters” during the extended incubation period of up to 14 days [5].

Vaccines are a highly efficient means of disease prevention in general, and as such, there has been a great focus on the shortening of their development pipeline. As the pandemic lingers on, new SARS-CoV-2 variants emerge with altered biological behavior and surprising fitness (e.g., Delta variant B.1.671.2 or Omicron B.1.1.529) [6,7,8,9]. The preventive potential of vaccination against COVID-19 with severe progress is high but limited in terms of SARS-CoV-2 transmission [10]. As no single vaccination program against respiratory viral infection offers complete population-wide protection, due to a large number of variables, including generation of novel variants outside of current vaccination coverage, the demand for novel antivirals for containment of coronavirus infections remains high [11,12]. 

In 2020, Signer et al. published in vitro data revealing a broad virucidal activity for *Echinacea purpurea* (hydroethanolic extract (65% *v*/*v*) of freshly harvested *Echinacea purpurea* (L.) Moench (95% aerial parts and 5% root) in pharmaceutical quality according to good manufacturing practices (GMP)) against a broad number of coronaviruses ranging from the typical common cold CoV-229E to highly pathogenic SARS-CoV-2 viruses [13]. The clinical relevance of the findings, however, remained uncertain, because direct contact of the pathogen with the extract was a prerequisite for full activity, although this was considered possible by sucking *Echinacea purpurea* tablets and/or gargling tincture in the pharynx.

In addition to direct virucidal and antiviral capacity, *Echinacea* extracts influence the immune system in a manner best described as adaptive immune modulation, rather than pure immune stimulation. *Echinacea* significantly reduced expression of inflammatory cytokines tumor necrosis factor TNF-α and interleukin IL-1-β by up to 24% compared to baseline, and increased levels of the anti-inflammatory cytokine IL-10. Additionally, there was an increase of up to 50% in the production of the immune-response modulating and antiviral interferon IFN-γ [14,15]. Further immunomodulatory mechanisms of *Echinacea* were shown to involve potent activation of the endocannabinoid system (ECS) by specific N-alkylamides via the cannabinoid receptor type 2 (CB2) [16,17]. Several of these bioactive N-alkylamides are structurally similar to endocannabinoids (e.g., anandamide), which influence the cytokine milieu in an anti-inflammatory manner at low nanomolar concentrations [18]. During COVID-19 progression, activation of the endocannabinoid system (ECS) could be an additional approach against systemic inflammation and the cytokine storm [19]. *Echinacea purpurea* could therefore reduce inflammatory processes through synergistic immunopharmacological targeting of CB receptors, mild inhibition of the fatty acid amide hydrolase (FAAH), or endocannabinoid transport [20,21]. In addition to immunoinflammatory changes, during respiratory viral infections, macrophages and neutrophils produce numerous reactive oxygen species (ROS) including hydrogen peroxide (H_2_O_2_), superoxide (O_2_^−^), and hydroxyl (OH) radicals, which institute inflammation and cell death in multiple organs, including the lungs [22].

Preclinical studies thus attribute pharmacological actions to *Echinacea*, which could be beneficial for the prevention and treatment of viral respiratory diseases. The goal of this review was to identify clinical studies assessing coronavirus infections in the context of *Echinacea* administration and to evaluate preventive and treatment benefits.

## 2. Materials and Methods

We searched the available literature on MEDLINE and EMBASE from inception up to 23rd March 2021 limited to human studies and included all synonyms of the following Medical Subject Headings MeSH terms: *Echinacea* species OR coronavirus OR virus infections AND Roter Sonnenhut or Purple Coneflower OR *Echinacea purpurea* OR human clinical studies OR respiratory tract infections AND terms related to randomized controlled trial (RCT) study design. A detailed search strategy is shown in the Supplementary Materials.

Obtained articles were further selected for clinical trials studying respiratory tract infections in humans and detection of respiratory viruses independently by SN and GG. In case of discrepancy regarding eligibility of articles, consensus was sought between all authors prior to inclusion or rejection. The research outcome was to be presented in a flow chart as requested by the preferred reporting items for systematic reviews and meta-analyses (PRISMA) [23]. In case of inconsistencies or missing information, the corresponding authors of the published article were contacted for further information. European and US clinical trials registers, EudraCT, and clinicaltrials.gov were screened for clinical studies on *Echinacea* and respiratory tract infections and researchers contacted for possible results.

Data extraction was undertaken using a pre-defined form retrieving the following information: primary author’s name, publication date, country, study design, sample size, researched plant species and preparation, duration of treatment (prevention vs. acute treatment), dosage, incidence of coronavirus virus infections and virus concentration (Cycle threshold (Ct-values), indicative of viral load as determined by RT-PCR), information regarding symptomatic development (e.g., total symptom score (TSS), episode duration, area-under-curve (AUC) or similar). The methodological quality of included studies was assessed using the criteria as proposed by Jadad [24]. Results are provided for safety (SAF), intention-to-treat (ITT), or as indicated for the per protocol (PP) populations.

## 3. Results

A total of N = 1687 articles were identified through database searching including PubMed and EMBASE using a combination of keywords, including MeSH/Emtree terms (Embase Subject Headings). For further details see Supplementary Materials.

**Figure 1:**
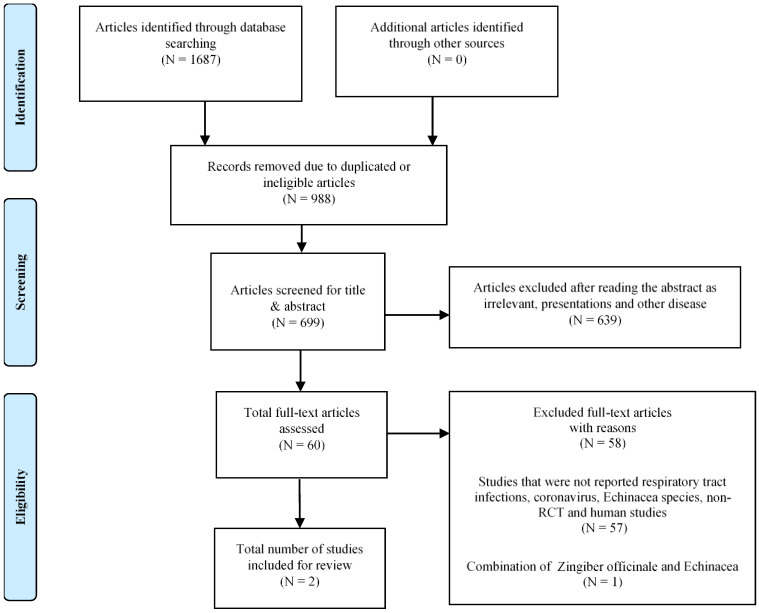
PRISMA flow chart for systematic reviews detailing the database searches, the number of publications screened, and eligible results retrieved.

N = 988 articles were excluded at the initial screening due to duplicated and irrelevant articles. Sixty potentially relevant articles were selected for full text assessment, of which 58 were further excluded due to studies that were not reporting respiratory tract -or coronavirus infections, *Echinacea* species, non-RCT and human studies (N = 57) or combination of Zingiber officinale and *Echinacea* (N = 1), which is detailed in the PRISMA flow chart (Figure 1). Finally, two studies meeting the eligibility criteria were selected for the systematic review (Table 1).

Due to the low number of referenced studies, we abstained from carrying out a quantitative meta-analysis. Instead, we decided to qualitatively discuss the observed findings from the two contributions, which are detailed in the following.

A first clinical study was carried out by Jawad et al. at Cardiff University, United Kingdom as a double-blinded, placebo-controlled, monocentric RCT [25]. Over a period of 4 months, participants from Cardiff, Wales, UK over 18 years were to apply a solution of 3 × 0.9 mL daily. The hydro-ethanolic extract (57.3% m/m) from freshly harvested aerial parts of *Echinacea purpurea* to 95% supplemented with 5% *Echinacea purpurea* roots extract was used from a commercial preparation (Echinaforce^®^ drops, A.Vogel AG, Roggwil, Switzerland, licensed there as a phytopharmaceutical), standardized to contain 5 mg/100 g of dodecatetraenoic acid isobutylamide, based on high-performance liquid chromatography measurements. It corresponded to the standard adult dosage of 2400 mg/d of the spissum extract from the same manufacturer, of which 1200 mg/d were used as recommended dosage for children in the second trial. The placebo solution was similar in shape, color, consistency, odor, flavor, and amount of alcohol. Patients were advised to keep the remedy in the mouth for 10 s prior to swallowing. The study looked at the occurrence of cold days and respiratory tract episodes as defined by Jackson et al. and assessed symptoms via diary [27]. Alongside symptom recording, patients were to take one nasopharyngeal sample on day 3 of each episode for identification of the respiratory pathogen using xTag FAST Track Multiplex Panel (Luminex, Austin, TX, USA). Analysis was carried out by the Provincial Health Services Authorities PHSA; BC Center for Disease Control, Vancouver, Canada. The following pathogens were screened: Influenza A H1/H3, Influenza B, Respiratory Syncytial Virus, Coronavirus 229E/OC43/NL63/HKU1, Parainfluenza virus 1–4, human Metapneumovirus, Entero-rhinovirus, Adenovirus, and human Bocavirus. The methodological quality according to Jadad et al. [24] reached the maximum score of 5.

**Figure 2:**
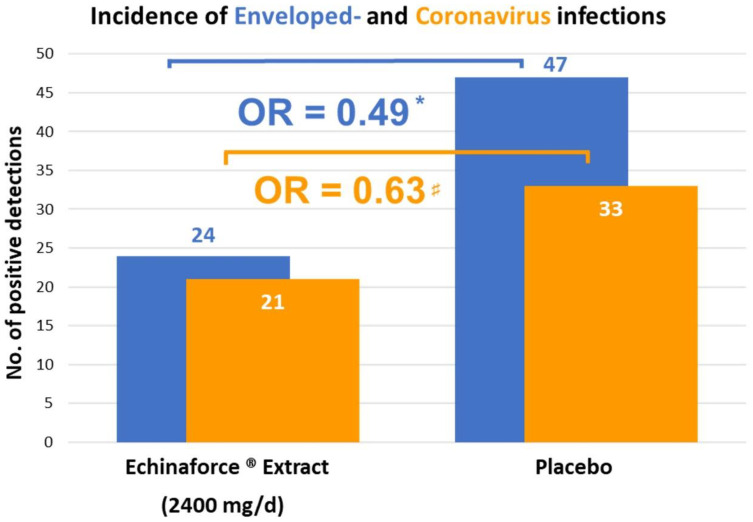
*Echinacea* significantly prevented enveloped virus infections (blue bar, * *p* = 0.0114), including coronaviruses 229E, HKU, NL63 and OC43 (orange bar, # *p* > 0.05), as measured by Jawad et al. 2012. Prevention of coronaviruses was significant for the ITT sub-group (*p* = 0.010).

Overall, N = 755 subjects were randomized to *E. purpurea* or placebo and N = 717 subjects were eligible for analysis as per safety collective (SAF). Preventive benefits were reported on the level of total cold episodes, cumulative cold days and recurrent episodes. The *Echinacea* group (N = 355) provided 86 nasopharyngeal samples in comparison to 115 samples from control group (N = 362), of which 54 and 74 samples, respectively, tested positive for any respiratory virus (*p* = 0.0663). In 24 and 47 samples, respectively, the presence of enveloped viruses was verified, and the resulting odds ratio OR = 0.49 calculated to be statistically significant (*p* = 0.0114, X^2^ test). The original publication, however, did not provide further information on coronavirus infections, which we retrieved from correspondence with authors of the study [25]. In the *Echinacea* group, 9 CoV-229E, 11 CoV-HKU1, and 1 CoV-OC43 infections were detected, for 21 coronavirus infections in total (overall incidence rate 5.9%). In comparison, in the placebo group, 15 CoV-229E, 17 CoV-HKU1, and 1 CoV-OC43 infections, totaling to 33 infections, were detected (overall incidence rate 9.1%). The resulting odds of contracting coronavirus infection were, with OR = 0.63, similar to the overall results on cumulated enveloped viruses (OR = 0.49; *p* = 0.0114, X^2^ test) (Figure 2). Preventive effects on coronaviruses were statistically significant in a collective of participants (*Echinacea* N = 163 and placebo N = 165), who had actively used their patient diary to report adverse events and/or cold-related symptoms (ITT sub-group analysis [28]). Here, an overall incidence rate of 5.5% for *Echinacea* vs. 14.6% for placebo was seen (*p* = 0.010, X^2^ test). Ct values of individual virus detections were not analyzed in this study and therefore conclusions regarding viral loads were not possible. Although fewer coronavirus infections were detected with *Echinacea* extract, the mean duration of episodes was, at 5.8 days [95% CI: 4.4–7.2] versus 5.9 days [95% CI: 4.4–7.5], unchanged.

In a second blinded, multi-center RCT, the same *Echinacea* extract formulated in tablets was given to children 4 to 12 years old living in central Switzerland [26]. Here, the authors chose low-dose vitamin C 3 × 50 mg/d as control. The commercial product was the child-friendly *Echinacea* formulation (Echinaforce^®^ Junior tablets, A. Vogel AG, Roggwil, Switzerland, also licensed as phytopharmaceutical drug) with a recommended dose of 1200 mg/d for children over 4 years. This trial enrolled 203 principally healthy children in the winter season of 2016/17, of which N = 103 were randomized to *Echinacea* 3 × 400 mg/d and N = 98 to Vitamin C 3 × 50 mg/d. The preparations were administered over a period of 4 months, including a 1-week treatment break after 2 months. Parents recorded and reported symptoms in their children using a validated scoring manual developed by Taylor and colleagues and collected a nasopharyngeal sample on day 2 of any episode [29]. The probe was sent for analysis using semi-quantitative RT-PCR (Allplex Respiratory Full Panel Assay, Seegene Inc., Seoul, South Korea), and the following pathogens were tested: rhinovirus, adenovirus, enteroviruses, respiratory syncytial virus, influenza and coronaviruses (229E, OC43 and NL63). The methodological quality according to Jadad et al. [24] was 4 points, which was again considered high.

**Figure 3:**
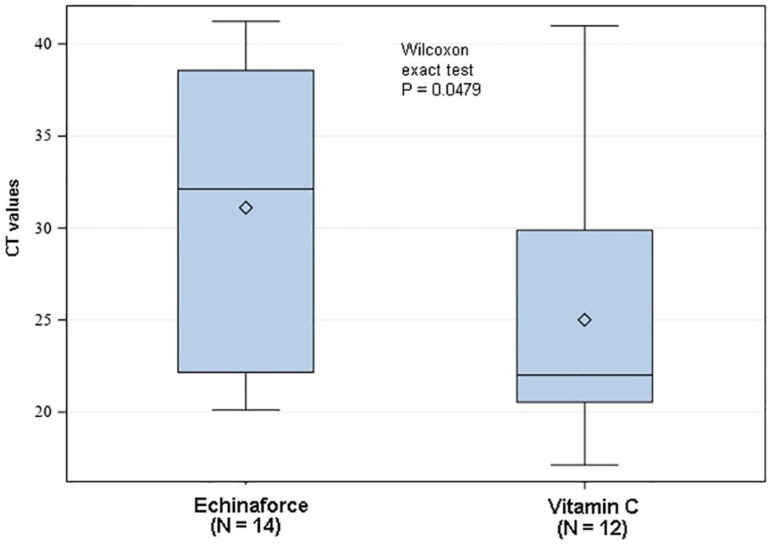
Preventive treatment of children with *Echinacea* reduced nasopharyngeal coronavirus concentrations significantly by 98.5% in comparison to control (Vitamin C, *p* < 0.05).

Overall, 57 and 72 samples were positively tested for ribonucleic acid (RNA) from any respiratory virus during prevention with *Echinacea* and control, respectively (*p* = 0.0074, X^2^ test). Similar to the results obtained from adults, *Echinacea* significantly reduced the incidence of enveloped virus infections in children with a total of N = 29 samples compared to control with N = 47 yielding a significant odds ratio of OR = 0.43 (*p* = 0.0038, X^2^ test). Again, information relating to coronavirus subtypes was retrieved from study authors retrospectively: coronavirus incidence was, at 13 and 11 positive samples in the intention-to-treat sample, insignificantly different between interventions (*p* > 0.1). Two CoV episodes in each group pre-existed at inclusion and were thus not regarded for prevention analysis, but for evaluation of treatment benefits. In contrast to the earlier study by Jawad et al., here, Ct-values were determined for respective incidence tests, estimating the number of RNA copies as a measure for virus concentration in the nasopharyngeal sample. The higher the Ct-value, the lower the viral load represented by the sample’s RNA level. *Echinacea* significantly increased the average Ct-value by 6.1 Ct units from 25.0 [95% CI: 20.5; 29.5] to 31.1 [95% CI: 26.3; 35.9], indicating a strong, −1.81 log or 98.5% reduction in coronavirus concentration (*p* = 0.0479, Wilcoxon test) relative to the control. Similar ∆Ct values of 5.53 and 11.92 were observed for variants NL63 and OC43, equaling a virus log reduction by −1.67 and −3.59 (Figure 3), corresponding to an absolute virus reduction of 97.8% and 99.97%.

Next, we analyzed the symptomatic development of confirmed coronavirus infections in children treated with *Echinacea* or control. To this end, an aggregated total symptom sum score was composed of ‘runny nose’, ‘blocked nose’, ‘sneezing’, ‘headache and aching limbs’, ‘sore throat’, ‘cough’, ‘chilliness’, ‘disturbed sleep quality of the child’, ‘malaise’, ‘need for additional care-giving’, each rated as ‘absent’, ‘mild’, ‘moderate’, ‘severe’, or ‘severity not assessable’. The AUC indicated a strong, 64.2% reduction from 75.8 [95% CI: 39.8–111.7] to 27.1 [95% CI: 14.8–39.4] score points in children supplemented with *Echinacea* (*p* = 0.0036). Results are provided for the intention-to-treat (ITT) collective, but did not relevantly change in the per protocol group (PP). Apparently, coronavirus infections during Echinaforce^®^ prevention were significantly less severe from the very first day of episode until the end of 10-day assessment.

## 4. Discussion

*Echinacea* has a long tradition for the prevention and the acute treatment of respiratory tract infections, and recent investigations attributed antiviral, immune-modulatory and anti-inflammatory pharmacological actions to the medicinal plant [14,20,30,31,32]. A wide series of respiratory viruses have been shown to be sensitive to lipophilic extracts of freshly harvested *Echinacea purpurea* and an obvious specificity towards enveloped pathogens could be verified [33]. The extract appears to block interaction of viral docking receptors (e.g., hemagglutinin on influenza) with structures on the target cell and is thus expected to prevent infection, although the detailed mechanism of action remains to be elucidated [31].

Antiviral effects on endemic coronaviruses causing typical cold symptoms (CoV-229E) and on highly pathogenic SARS-CoV-1 and MERS-CoV type have been studied in vitro for several years. Recently, these effects have also been confirmed for the newly occurring SARS-CoV-2 [13,34]. Signer et al. concluded a generally applicable principle of action against coronaviruses, but pointed out that clinical data are needed to confirm their in vitro findings. This work aimed to gather any evidence from in vivo activity against coronavirus infections from already published clinical studies.

Despite the large number of *Echinacea* clinical trials, we identified only two clinical studies that collected nasopharyngeal samples for virus testing [25,26]. Both studies accurately described blinding, randomization, were appropriately controlled and referred to data set from almost 1000 patients treated over a period of 4 months with either tincture or tablets from the same 65% (*v*/*v*) ethanolic extract from *Echinacea purpurea* (Echinaforce^®^). Due to the low number of included studies, we abstained from pooling results, rather opting to qualitatively describe outcomes from the studies.

Preventive antiviral effects of *Echinacea* with coronavirus infections were seen in both studies by varying parameters. Four-month supplementation with *Echinacea* in adults and children reduced the incidence of coronaviruses as part of its effect on enveloped virus infections and virus concentration in nasopharynx in the latter, respectively. Jawad did not measure virus concentrations (Ct-value), and this information was therefore lacking. Ogal employed a newer, more sensitive method for detection that was able to detect as few as 100 viral genome copies. We propose that *Echinacea*’s antiviral effects reduce virus replication, therefore reducing virus load below detection limits for standard PCR resulting in decreased incidence with *Echinacea* as observed in the Jawad study. This is consistent with the lower virus Ct-values observed in the Ogal study as even residual, subclinical viral concentrations could still be quantified.

Interestingly, prevention with *Echinacea* appeared to reduce the symptomatic development only in children rather than in adults. However, Jawad et al. did not sample continuously, but only upon symptomatic infection, and may have missed potential subclinical infections with verum for the above reasons, skewing evaluation at this level. In contrast, the low virus detection limit in Ogal et al. due to the use of RT-qPCR methods might have identified more milder/subclinical episodes which would have further reduced symptoms with *Echinacea* treatment in adults as observed for children. In this respect, incidence (detections) and symptomatology/Ct values must be considered as complementary parameters, both indicative for antiviral effects of *Echinacea*. Taking this into account, we conclude that preventive treatment with *Echinacea* provides beneficial effects to coronavirus infections in both, adults and children. In adults, infections could be prevented, whereas children demonstrated significantly reduced virus loads, symptom reduction, as well as shortened duration of illnesses. In both cases, preventive effects of *Echinacea* against coronaviruses could be ascertained in controlled clinical settings.

Neither of the referenced studies detected the newly occurring SARS-CoV-2, but only endemic coronavirus strains, as both were carried out prior to 2019. Conclusions pertaining to COVID-19 must be carefully considered, but are nevertheless important, since many people rely on immune support by herbal preparations these days, and it is important to give an estimation on evidence.

All coronaviruses share structural similarities, including an enveloped membrane containing the genetic material, i.e., RNA and spike proteins for attachment to target cells. *Echinacea* broadly inhibits enveloped respiratory viruses at physiological concentrations in vitro and in vivo; however, the exact mode-of-action remains to be elucidated. Due to this broad range of activity, deactivation of all measured coronavirus types, such as alpha (CoV-229E) and beta strains (MERS-CoV, SARS-CoV-1, SARS-CoV-2) by *Echinacea*, can realistically be assumed, and the results of this study point towards the clinical relevance of in vitro findings. Though not always reaching statistical significance for the particular virus, the effects of *Echinacea* against coronaviruses are mirrored and overarched by the effects seen against membranous viruses.

In addition, a recently completed clinical study was also identified on clinicaltrials.gov (ID: NCT05002179), investigating an *Echinacea purpurea* preparation from fresh herbs/roots at dosages of 2400 mg–4000 mg/d extract per day over 5 months in comparison with non-treatment. The same preparation as in Ogal et al. was used, but at the recommended posology for adults. The study was carried out in N = 120 adults from November 2020 until May 2021 and routinely collected naso-/oropharyngeal/blood samples for detection of respiratory virus infections, including SARS-CoV-2. The results suggest a significantly reduced risk for SARS-CoV-2 infections, measured either as symptomatic COVID-19 illness, RT-PCR positive sample, or by seroconversion. Summarized over all phases of prevention, 21 and 29 samples tested positive for any virus in the *Echinacea* and control group, of which 5 and 14 samples tested SARS-CoV-2 positive, respectively (RR = 0.37, X^2^ test, *p* = 0.03). Overall, 10 and 14 symptomatic episodes occurred, of which 5 and 8 were COVID-19 (RR = 0.70, X^2^ test, *p* > 0.05). *Echinacea* treatment, when applied during acute episodes, significantly reduced the overall virus load by at ~99% (2.12 log10, *t*-test, *p* < 0.05), as well as the “time to virus clearance” by 8.0 days for all viruses (Wilcoxon test, *p* = 0.02) and by 4.8 days for SARS-CoV-2 (*p* > 0.05) in comparison to control. Additionally, *Echinacea* treatment significantly reduced fever days (1 day vs. 11 days, X^2^ test, *p* = 0.003) [35].

These findings are still awaiting publication in a peer-reviewed journal, and need to be treated with appropriate caution. They were nevertheless mentioned in the current review for the sake of completeness and because they seem to confirm the applicability of the antiviral benefits of *Echinacea* to a broad range of coronaviruses, including SARS-CoV-2.

Despite the limitations associated with this review (e.g., low number of studies), we believe that our findings are highly relevant, as they provide a rather consistent picture on antiviral and preventive benefits of *Echinacea* in coronavirus infections overall. Additionally, they have important implications for the preventive use of *Echinacea* during COVID-19 epidemic. The reduction of coronavirus loads is medically highly relevant as virus concentrations appear to correlate with community transmission, influence illness severity and progression in adults and children [36,37,38,39]. Two clinical studies have shown over 98.5% reduction of coronavirus concentration in nasal secretions obtained from children and adults treated with *Echinacea*. Evidence is still missing that vaccines significantly reduce viral loads, especially of SARS-CoV-2 delta variant and this asset would be a decisive argument for use of *Echinacea* during COVID-19 pandemic.

Notably, all cited studies applied the same *Echinacea purpurea* extract (Echinaforce^®^) either as diluted tincture or as tablets, both of which were kept in the mouth for a while prior to swallowing. As already hypothesized by Signer et al., pharyngeal administration of the extract may be key to inactivating respiratory viruses at the main entry site prior to infection [13]. Furthermore, any viral attenuation in this region could prevent further dissemination of nasal infections to the lungs, as seen with severe progressions of COVID-19. Indeed, a recent meta-analysis found a significant prevention of pneumonia secondary to viral respiratory tract infections upon 2 to 4 months of *Echinacea* preventive use [40].

## 5. Conclusions

A specific extract of *Echinacea purpurea* (L.) Moench exhibits direct antiviral activity against a broad range of respiratory pathogens, including coronaviruses. This extract supports the tonic production of IFN-γ and beneficially modulates inflammatory cytokines like TNF-α. Two RCTs with phytopharmaceutical preparations of this extract demonstrate effective protection against enveloped viruses including coronaviruses in adults and children. Preliminary published clinical results on SARS-CoV-2 may yet further support the use of *Echinacea* also against this particular coronavirus.

## Figures and Tables

**Table 1:** Included studies with details on methodology and outcome.

Study	Population	Country	Jadad Score	Plant Species	Preparation	Dosage	Control	Sample Source	Analysis Method	Symptomatic Assessments
Jawad M, et al. 2012 [25]	Adults > 18 years, N = 755	UK	5	*Echinacea purpurea*	Alcoholic extract of freshly harvested herb/roots	3 × 20 drops/d	Placebo	Naso- pharynx	RT-PCR	Individual and TSS, AUC
Ogal M, et al. 2021 [26]	Children 4–12 years, N = 203	CH	4	*Echinacea purpurea*	Alcoholic extract of freshly harvested herb/roots	3 × 1 tablet/d	Vitamin C	Naso- pharynx	RT-PCR, Ct-values reported	Individual and TSS, AUC

## Supplementary Materials

The following supporting information can be downloaded at: https://www.mdpi.com/article/10.3390/microorganisms10020211/s1, The search strategy and results are detailed in the supplementary materials.
