# Supplementary material for: Echinacea as a Potential Force against Coronavirus Infections? A Mini-Review of Randomized Controlled Trials in Adults and Children
Source: Microorganisms. 2022 Jan 19;10(2):211. doi: 10.3390/microorganisms10020211 (PMC8879308; doi:10.3390/microorganisms10020211)

Appendix 1

Study Title

Echinacea as a Potential Force against Coronavirus Infections? A Mini-Review of Randomized Controlled Trials in Adults and Children

Aim

The goal of this review was to identify coronavirus infections in clinical studies on Echinacea and to evaluate preventive and treatment benefits.

Methods

The Preferred Reporting Items for Systematic Reviews and Meta-Analyses (PRISMA) guidelines were followed to conduct and write this review [1].

Eligibility Criteria for Study Selection

Eligible studies were selected based on the following criteria:

(i) studies reporting original data on effect of Echinacea on respiratory tract pathogens

(ii) studies were analyzed if they reported human sampling and assessed the effect of prevention or therapy with *Echinacea species* such as *Echinacea* or *Echinacea purpurea* or *Echinacea* or coneflower, which have been identified as playing a role in evidence for in vivo and in vitro activity against coronavirus infections

(iii) we included randomized controlled trials (RCTs), clinical studies, which assess the effect of Echinacea on respiratory tract pathogens in humans. Abstracts and unpublished studies were not included. When articles contained potentially relevant data not fully reported, the authors were contacted by email for further information.

Outcomes measures

Data extraction was undertaken using a pre-defined form retrieving the following information: primary author’s name, publication date, country, study design, sample size, researched plant species and preparation, duration of treatment (prevention vs acute treatment), dosage,

incidence of coronavirus virus infections and virus concentration (Ct-values), information regarding symptomatic development (e.g. total symptom score, episode duration, area-under-curve or similar). The methodological quality of included studies was assessed using the criteria as proposed by Jadad [2].

Search Strategy

We used PubMed and EMBASE databases for the literature search using a combination of keywords, which includes Medical Subject Headings (MeSH)/*Emtree* terms (Embase Subject Headings). The search strategy was limited to English, humans and included all synonyms of the following MeSH terms: Echinacea species OR inclusion coronavirus virus infections, AND Roter Sonnenhut or Purple Coneflower OR Echinacea purpurea OR human Clinical Studies OR Respiratory Tract Infections AND terms related to study design (RCTs). The following search headings/ MeSH terms were used:

(Randomized Controlled Trials as Topic / OR randomized controlled trial/ OR Random Allocation/ OR Double Blind Method/ OR Single Blind Method/ OR clinical trial/ OR clinical trial, phase OR clinical trial, phase ii.pt. OR clinical trial, phase iii.pt. OR clinical trial, clinical studies OR phase iv.pt. OR controlled clinical trial.pt. OR randomized controlled trial.pt. OR multicenter study OR Placebo OR randomly allocated.)

AND (“Echinacea species” (MeSH Terms) OR “Echinacea species” [All Fields]) OR (“Purple Coneflower” (MeSH Terms) OR “Purple Coneflower” [All Fields]) OR (“Echinacea purpurea ” (MeSH Terms) OR “Echinacea purpurea ” [All Fields]) OR (“Echinacea [All Fields] AND “purpurea [All Fields]) OR (“Echinacea species, Roter sonnenhut ” (All Fields) OR (“Echinacea species”(All Fields) AND “Roter”(All Fields) AND “sonnenhut”(All Fields))

AND ("clinical study"[Publication Type] OR "clinical studies as topic"[MeSH Terms] OR "clinical studies"[All Fields]) AND ("respiratory tract infections"[MeSH Terms] OR ("respiratory"[All Fields] AND "tract"[All Fields] AND "infections"[All Fields]) OR "respiratory tract infections"[All Fields]) AND ("human s"[All Fields] OR "humans"[MeSH Terms] OR "humans"[All Fields] OR "human"[All Fields]) ("respiratory system"[MeSH Terms] OR ("respiratory"[All Fields] AND "system"[All Fields]) OR "respiratory system"[All Fields] OR ("respiratory"[All Fields] AND "tract"[All Fields]) OR "respiratory tract"[All Fields]) AND ("virology"[MeSH Subheading] OR "virology"[All Fields] OR "viruses"[All Fields] OR "viruses"[MeSH Terms] OR "virus s"[All Fields] OR "viruse"[All Fields] OR

"virus"[All Fields]) AND ("body fluids"[MeSH Terms] OR ("body"[All Fields] AND "fluids"[All Fields]) OR "body fluids"[All Fields])

AND “Respiratory Tract Infections” (MeSH Terms) OR (“Respiratory” [All Fields] AND “Tract [All Fields] AND “Infections” [All Fields]) OR “Respiratory Tract Infections” {All Fields] OR (“Respiratory” [All Fields} AND “Tract” [All Fields] AND “Infections” [All Fields]) OR “Respiratory Tract Infections” [All Fields]) “safety and efficacy ” (MeSH Terms) OR (“safety” [All Fields] AND “efficacy” [MeSH Terms] AND “common cold” (MeSH Terms) OR (“common” [All Fields] AND “cold” [MeSH Terms] OR "disease"[All Fields] OR "diseases"[All Fields] OR "disease s"[All Fields] OR "diseased"[All Fields]) “corona virus” (MeSH Terms) OR (“corona” [All Fields] AND “Virus” [All Fields]) [2] [‘SARS-CoV2)OR ‘SARS’ OR ‘CoV2’] AND [children OR paediatric] AND [adults].

Data Extraction

After applying the eligibility criteria, articles that were relevant for full-text screening were chosen. Two authors worked independently on article screening and data extraction. If there were any disagreements, they were settled by discussion or consensus with a third independent reviewer. The articles were initially screened on the basis of their title, followed by the abstract of the article. The title and abstract of the articles were irrelevant to the present investigation; these were excluded from the secondary screening. The selected articles from the initial screening were assessed for full-text screening to find out the eligibility criteria of the current review.

Results

A total of 1687 articles were identified through database searching including PubMed and EMBASE, of which 988 articles were excluded at the initial screening due to duplicated and irrelevant articles. 60 potentially relevant articles were selected for the full text assessment, of which 58 were further excluded due to studies that were not reported respiratory tract infections, coronavirus, Echinacea species, non-RCT and human studies (n= 57) and combination of Zingiber officinale and Echinacea (n= 1), which was detailed in PRISMA flow chart (Figure 1). Finally, 2 articles were selected for the narrative review, which met the eligibility criteria of the current study.

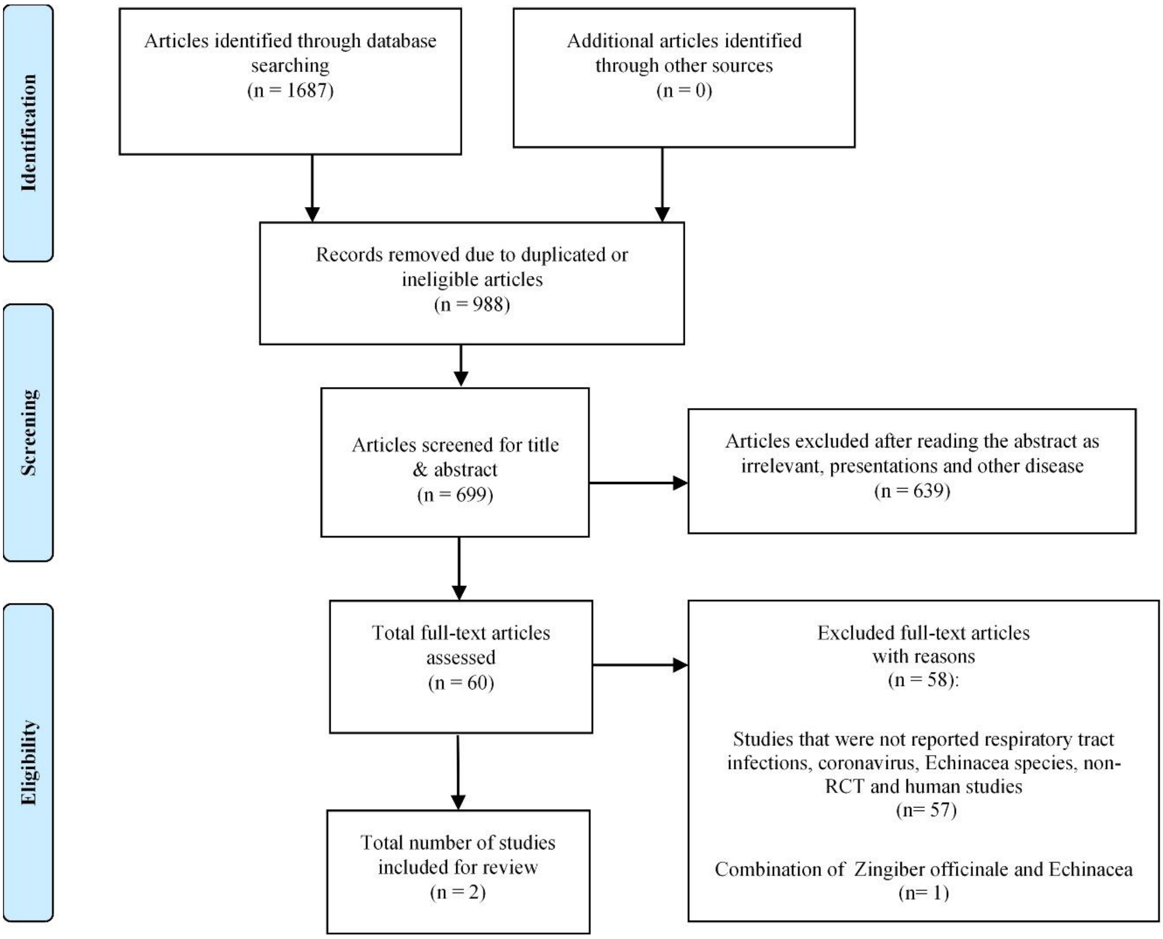

Figure S1: PRISMA flow chart for systematic reviews detailing the database searches, the number of publications screened and eligible results retrieved.

References

1. Moher, D.; Liberati, A.; Tetzlaff, J.; Altman, D.G. Preferred reporting items for systematic reviews and meta-analyses: the PRISMA statement. *PLoS Med.* **2009**, *6*, e1000097, doi:10.1371/journal.pmed.1000097.
2. Jadad, A.R.; Moore, R.A.; Carroll, D.; Jenkinson, C.; Reynolds, D.J.; Gavaghan, D.J.; McQuay, H.J. Assessing the quality of reports of randomized clinical trials: is blinding necessary? *Control. Clin. Trials* **1996**, *17*, 1–12, doi:10.1016/0197-2456(95)00134-4.

Annex

A1. Eligibility criteria

A2. Search strategy

A3. Database specific strategies

A4. Search strategy

A5. Included and excluded articles with justification

A6. Data extraction template

A1 Eligibility criteria.

Table A1 Eligibility criteria.

**Inclusion criteria**	**Exclusion criteria**
Randomized controlled trials<br><br>Studies that reported the safety and efficacy of Echinacea species against respiratory tract pathogens<br><br>Studies that reported the safety and efficacy of Echinacea species against coronavirus<br><br>Children and adults population	Conference proceedings, editorials letters and other study designs (Non-RCT).<br><br>Studies that were used other than Echinacea species<br><br>Studies that did not evaluated the respiratory tract pathogens especially corona virus

A2 Search strategy

Table A2 Keywords included in the search strategy for all PubMed and Embase databases; terms searched for in the title and abstract of papers.

**Condition**	Echinacea **OR** Echinacea purpurea **OR** Roter Sonnenhut **OR** Purple Coneflower<br><br>**AND**<br><br>“Respiratory Tract Infections” **OR** “common cold ” **OR** “Respiratory Tract Pathogens” **OR** “coronavirus”
	**AND**
**Study type term**	Randomized Controlled Trials or Double-blind or Single-blind or Random allocation or Clinical trial or Clinical studies<br><br>**AND**<br><br>Humans **OR** Adults **OR** Children

A3 Database specific search strategies

A3.1 PubMed Search Strategy – limit to human,

((Randomized Controlled Trials as Topic / OR randomized controlled trial/ OR Random Allocation/ OR Double Blind Method/ OR Single Blind Method/ OR clinical trial/ OR clinical trial, phase OR clinical trial, phase ii.pt. OR clinical trial, phase iii.pt. OR clinical trial, phase iv.pt. OR controlled clinical trial.pt. OR randomized controlled trial.pt. OR multicenter study OR Placebo OR randomly allocated.)

AND (“Echinacea species” (MeSH Terms) OR “Echinacea species” [All Fields]) OR (“Purple Coneflower” (MeSH Terms) OR “Purple Coneflower” [All Fields]) OR (“Echinacea purpurea ” (MeSH Terms) OR “Echinacea purpurea ” [All Fields]) OR (“Echinacea [All Fields] AND “purpurea [All Fields]) OR (“Echinacea species, Roter sonnenhut ” (All Fields) OR (“Echinacea species”(All Fields) AND “Roter”(All Fields) AND “sonnenhut”(All Fields)) AND ("clinical study"[Publication Type] OR "clinical studies as topic"[MeSH Terms] OR "clinical studies"[All Fields]) AND ("respiratory tract infections"[MeSH Terms]

AND “Respiratory Tract Infections” (MeSH Terms) OR (“Respiratory” [All Fields] AND “Tract [All Fields] AND “Infections” [All Fields]) OR “Respiratory Tract Infections” {All Fields] OR (“Respiratory” [All Fields} AND “Tract” [All Fields] AND “Infections” [All Fields]) OR “Respiratory Tract Infections” [All Fields]) “safety and efficacy ” (MeSH Terms) OR (“safety” [All Fields] AND “efficacy” [MeSH Terms] AND “common cold” (MeSH Terms) OR (“common” [All Fields] AND “cold” [MeSH Terms] OR "disease"[All Fields] OR "diseases"[All Fields] OR "disease s"[All Fields] OR "diseased"[All Fields]) “corona virus” (MeSH Terms) OR (“corona” [All Fields] AND “Virus” [All Fields]) [2] [‘SARS-CoV2)OR ‘SARS’ OR ‘CoV2’]

A3.2. EMBASE Search Strategy – limit to human,

Echinacea species’/exp OR ‘Echinacea species’ :ab,ti OR ‘Echinacea purpurea ’ /exp OR ‘Echinacea purpurea ’: ab, ti OR ‘Echinacea purpurea ’/exp OR ‘Purple Coneflower’/ exp OR ‘Purple Coneflower’ :ab,ti OR ‘Purple Coneflower’ / exp OR ‘Purple Coneflower’ / exp OR exp Echinacea species / OR ‘Roter sonnenhut’ OR Echinacea purpurea.mp OR Purple Coneflower.mp OR Purple Coneflower.mp OR PC.mp AND (Respiratory Tract Infections /exp OR common, cold.mp or common cold /exp OR Respiratory Tract * Infections. ti,ab. OR common cold. ti,ab OR Respiratory Tract Infections.mp. or exp Respiratory Tract Infections / OR common cold.mo. or exp common cold /) AND (Incidence.mp. or exp safety/ OR efficacy.mp or exp efficacy OR epidemiological data/ OR disease progression/ OR disease activity/ OR ‘Randomized Controlled Trials *.tw’ OR ‘Random Allocation.tw’ OR ‘Double Blind’ OR ‘Randomized Controlled Trials.tw’ OR ‘clinical studies’ OR ‘clinical trial.tw’ or Placebo Controlled Trials /) AND Limit to (english language, human AND conference abstract AND conference paper AND conference proceeding AND “conference review”) AND yr=”

A4 Search results

PubMed- Search results

#	PubMed	
**Search**	**Keyword**	**Results**
1	Tier 1: Echinacea Purpurea	1,331
2	((Echinacea) OR (Roter sonnenhut)) OR (Purple cone flower)	1,340

3	Echinacea	1,338
4	Roter sonnenhut	0
5	Purple cone flower	8
6	(Echinacea Purpurea) AND (Respiratory Tract Infections)	203
7	((Echinacea Purpurea) AND (Respiratory Tract Infections)) AND (Randomised controlled trail)	0
8	(((Respiratory tract infections)) OR (common cold)) AND (Echinacea)	231
9	(Respiratory Tract Infections) AND (Echinacea)	203
10	(Echinacea) AND (common cold)	155
11	(((safety and efficacy)) AND (Respiratory Tract Infections)) AND (Echinacea)	19
12	(Echinacea) AND (safety and efficacy)	65
13	(Randomized controlled trail) AND (Echinacea)	0
14	(((Randomized controlled trail) OR (Double blind)) OR (single blind)) OR (clinical trial)	1,308,606
15	(Randomized Controlled Trial) AND (Safety and efficacy)	53,404
16	(((Randomized Controlled Trial) OR (single blind)) AND (clinical trial)) AND (Echinacea Purpurea)	109
17	((((((((Randomized controlled trail) OR (Double blind))) OR (single blind)) OR (clinical trial)) AND (Echinacea)) OR (Roter sonnenhut)) OR (Purple cone flower)	194
18	(Randomized controlled trial) AND (Respiratory Tract Infections)	14,460
19	Respiratory tract infections	493,848
20	((Clinical studies) AND (respiratory tract infections)) AND (humans)	31,002
21	(((Respiratory tract infections) AND (humans)) AND (Echinacea)) AND (clinical studies)	83
22	(Respiratory tract viruses) AND (body fluids)	1,279
23	((Echinacea) AND (respiratory tract viruses)) AND (body fluids)	0
24	(((respiratory tract viruses) AND (body fluids)) OR (Roter sonnenhut)) OR (Purple cone flower)	1,287
25	((Randomized controlled trial) AND (Echinacea)) AND (Respiratory Tract Infections)	57

Embase- Search results

#	EMBASE (2011-2021)	
**Search**	**Keyword**	**Results**
1	Tier 1: Echinacea Purpurea $	1,338
2	Echinacea OR Roter sonnenhut $ OR Purple cone flower	1,350
3	Echinacea $	1,344
4	Roter sonnenhut $	0
5	Purple cone flower $	11
6	Echinacea Purpurea AND Respiratory Tract Infections $	218
7	Echinacea Purpurea $ AND Respiratory Tract Infections AND Randomised controlled trail $	0
8	Respiratory tract infections OR common cold $ AND Echinacea	229
9	Respiratory Tract Infections $ AND Echinacea	218
10	Echinacea $ AND common cold	162
11	safety and efficacy $ AND Respiratory Tract Infections AND Echinacea $	18
12	Echinacea AND Safety and efficacy $	69
13	Randomized controlled trail $ AND Echinacea	0
14	Randomized controlled trail OR Double blind $ OR single blind OR clinical trial $	1,313,596
15	Randomized Controlled Trial $ AND Safety and efficacy	54,398
16	Randomized Controlled Trial OR single blind $ AND clinical trial AND Echinacea Purpurea $	112
17	Randomized controlled trail $ OR Double blind $ OR single blind OR clinical trial $ AND Echinacea OR Roter sonnenhut $ OR Purple cone flower	198
18	Randomized controlled trial AND Respiratory Tract Infections	13,260
19	Respiratory tract infections $	494,851
20	Clinical studies $ AND respiratory tract infections AND humans $	31,212
21	Respiratory tract infections AND humans $ AND Echinacea AND clinical studies$	87
22	Respiratory tract viruses $ AND body fluids	1,286
23	Echinacea AND respiratory tract viruses $ AND body fluids	0

24	Respiratory tract viruses AND body fluids$ OR Roter sonnenhut $ OR Purple cone flower	1,278
25	Randomized controlled trial AND Echinacea$ AND Respiratory Tract Infections	59

A5: Included and excluded articles with justification

S.NO	Author	Year	Title	Reason for exclusion and inclusion	Links
1	Motahareh Boozari et al	2021	Natural products for Corona virus infection prevention and treatment regarding to previous coronavirus infections and novel studies.	Excluded because NO evidence of randomised control trail	https://pubmed.ncbi.nlm.nih.gov/32985017/
2	Tibebeselassie Seyoum Keflie et al	2021	Micronutrients and bioactive substances: Their potential roles in combating Corona virus infection	Excluded because NO evidence of randomised control trail	https://pubmed.ncbi.nlm.nih.gov/33450678/
3	Andreas Hensel et al	2020	Challenges at the Time of Corona virus infection: Opportunities and Innovations in Antivirals from Nature	Excluded because NO evidence of randomised control trail	https://pubmed.ncbi.nlm.nih.gov/32434254/
4	Johanna Signer et al	2020	In vitro virucidal activity of Echinaforce®, an Echinacea purpurea preparation, against coronaviruses, including common cold coronavirus 229E and SARS-CoV-2	Excluded because NO evidence of randomised control trail	https://pubmed.ncbi.nlm.nih.gov/32907596/
5	Shaden A M Khalifa et al	2021	Screening for natural and derived bio-active compounds in preclinical and clinical studies: One of the frontlines of fighting the coronaviruses pandemic	Excluded because NO evidence of randomised control trail	https://pubmed.ncbi.nlm.nih.gov/33067112/

6	M F Nagoor Meeran et al	2021	Can *Echinacea* be a potential candidate to target immunity, inflammation, and infection - The trinity of coronavirus disease 2019	Excluded because NO evidence of randomised control trail	https://pubmed.ncbi.nlm.nih.gov/33585706/
7	Shiv Bharadwaj et al	2021	Structure-Based Identification of Natural Products as SARS-CoV-2 M pro Antagonist from *Echinacea angustifolia* Using Computational Approaches	Excluded because NO evidence of randomised control trail	https://pubmed.ncbi.nlm.nih.gov/33672054/
8	Marlies Karsch-Völk et al	2014	Echinacea for preventing and treating the common cold	Excluded because NO evidence of Corona virus infection	https://pubmed.ncbi.nlm.nih.gov/24554461/
9	Sholto David et al	2019	Echinacea for the prevention and treatment of upper respiratory tract infections: A systematic review and meta-analysis	Excluded because NO evidence of Corona virus infection	https://pubmed.ncbi.nlm.nih.gov/31126553/
10	Giulia Dante et al	2014	Herbal therapies in pregnancy: what works?	Excluded because NO evidence of Corona virus infection	https://pubmed.ncbi.nlm.nih.gov/24535321/
11	Songie Choi et al	2017	A systematic review of the pharmacokinetic and pharmacodynamic interactions of herbal medicine with warfarin	Excluded because NO evidence of Corona virus infection	https://pubmed.ncbi.nlm.nih.gov/28797065/
12	Aaron S Griffin et al	2018	Alternative therapies for chronic rhinosinusitis: A review	Excluded because NO evidence of Corona virus infection	https://pubmed.ncbi.nlm.nih.gov/29554408/

13	Attila Oláh et al	2017	Echinacea purpurea-derived alkylamides exhibit potent anti-inflammatory effects and alleviate clinical symptoms of atopic eczema	Excluded because NO evidence of Corona virus infection	https://pubmed.ncbi.nlm.nih.gov/28610718/
14	Philippe Vorilhon et al	2019	Efficacy of vitamin C for the prevention and treatment of upper respiratory tract infection. A meta-analysis in children	Excluded because NO evidence of Corona virus infection	https://pubmed.ncbi.nlm.nih.gov/30465062/
15	Ramon Weishaupt et al	2020	Safety and Dose-Dependent Effects of Echinacea for the Treatment of Acute Cold Episodes in Children: A Multicenter, Randomized, Open-Label Clinical Trial	Excluded because NO evidence of Corona virus infection	https://pubmed.ncbi.nlm.nih.gov/33333722/
16	Andreas Schapowal et al	2015	Echinacea reduces the risk of recurrent respiratory tract infections and complications: a meta-analysis of randomized controlled trials	Excluded because NO evidence of Corona virus infection	https://pubmed.ncbi.nlm.nih.gov/25784510/
17	Mehdi Safarabadi et al	2017	Comparing the Effect of Echinacea and Chlorhexidine Mouthwash on the Microbial Flora of Intubated Patients Admitted to the Intensive Care Unit	Excluded because NO evidence of Corona virus infection	https://pubmed.ncbi.nlm.nih.gov/29184589/

18	M Jawad et al	2012	Safety and Efficacy Profile of Echinacea purpurea to Prevent Common Cold Episodes: A Randomized, Double-Blind, Placebo-Controlled Trial	Included	https://pubmed.ncbi.nlm.nih.gov/23024696/
19	Surabhi Sinha et al	2015	Immunomodulators in warts: Unexplored or ineffective?	Excluded because NO evidence of Corona virus infection and Echinacea species	https://pubmed.ncbi.nlm.nih.gov/25814698/
20	E Tiralongo et al	2012	Randomised, double blind, placebo-controlled trial of echinacea supplementation in air travellers	Excluded because NO evidence of Corona virus infection	https://pubmed.ncbi.nlm.nih.gov/22229040/
21	Andreas Schapowal et al	2013	Efficacy and safety of Echinaforce in respiratory tract infections	Excluded because NO evidence of Corona virus infection	https://pubmed.ncbi.nlm.nih.gov/23263637/
22	F Isbaniah et al	2011	Echinacea purpurea along with zinc, selenium and vitamin C to alleviate exacerbations of chronic obstructive pulmonary disease: results from a randomized controlled trial	Excluded because NO evidence of Corona virus infection	https://pubmed.ncbi.nlm.nih.gov/21062330/
23	Mehdi Mesri et al	2021	The effects of combination of Zingiber officinale and Echinacea on alleviation of clinical symptoms and hospitalization rate of suspected Corona virus infection outpatients: a randomized controlled trial	Excluded due to combination of Zingiber officinale and Echinacea and no evidence of respiratory tract infections	https://pubmed.ncbi.nlm.nih.gov/33787192/

24	József Haller et al	2013	The anxiolytic potential and psychotropic side effects of an echinacea preparation in laboratory animals and healthy volunteers	Excluded because NO evidence of Respiratory tract infections	https://pubmed.ncbi.nlm.nih.gov/22451347/
25	J Orengo et al	2012	Evaluating the efficacy of cinnamaldehyde and Echinacea purpurea plant extract in broilers against Eimeria acervulina	Excluded because NO evidence of Respiratory tract infections	https://pubmed.ncbi.nlm.nih.gov/21996002/
26	Osama G Abdel-Naby Awad et al	2019	Echinacea can help with Azithromycin in prevention of recurrent tonsillitis in children	Excluded because NO evidence of Corona virus infection and randomised control trails	https://pubmed.ncbi.nlm.nih.gov/32487336/
27	Mercedes Ogal et al	2021	Echinacea reduces antibiotic usage in children through respiratory tract infection prevention: a randomized, blinded, controlled clinical trial	Included	https://pubmed.ncbi.nlm.nih.gov/33832544/
28	Hannah Ayrle et al	2021	Effects of an oral hydro-ethanolic purple coneflower extract on performance, clinical health and immune parameters in calves	Excluded because NO evidence of Corona virus infection	https://pubmed.ncbi.nlm.nih.gov/34144282/
29	John Grbic et al	2011	A phase II trial of a transmucosal herbal patch for the treatment of gingivitis	Excluded because NO evidence of Corona virus infection and Echinacea species	https://pubmed.ncbi.nlm.nih.gov/21965490/

30	S Dabbou et al	2016	Rabbit dietary supplementation with pale purple coneflower. 1. Effects on the reproductive performance and immune parameters of does	Excluded because NO evidence of Corona virus infection and human study	https://pubmed.ncbi.nlm.nih.gov/26763800/
31	Noah Samuels et al	2012	Effect of an herbal mouth rinse in preventing periodontal inflammation in an experimental gingivitis model: a pilot study	Excluded because NO evidence of Corona virus infection and Echinacea species	https://pubmed.ncbi.nlm.nih.gov/22479786/
32	D Melchart et al	2000	Echinacea for preventing and treating the common cold	Excluded because NO evidence of Corona virus infection	https://pubmed.ncbi.nlm.nih.gov/10796553/
33	Francesca Toselli et al	2009	Echinacea metabolism and drug interactions: the case for standardization of a complementary medicine	Excluded because NO evidence of Corona virus infection	https://pubmed.ncbi.nlm.nih.gov/19427870/
34	M A Flannery et al	1999	From rudbeckia to echinacea: the emergence of the purple cone flower in modern therapeutics	Excluded because NO evidence of Corona virus infection	https://pubmed.ncbi.nlm.nih.gov/11623947/
35	Theophilus B Kwofie et al	2012	Respiratory viruses in children hospitalized for acute lower respiratory tract infection in Ghana	Excluded because NO evidence of Corona virus infection and Echinacea species	https://pubmed.ncbi.nlm.nih.gov/22490115/
36	María J Giuffrida et al	2014	Increased cytokine/chemokines in serum from asthmatic and non-asthmatic patients with viral respiratory infection	Excluded because NO evidence of Corona virus infection and Echinacea species	https://pubmed.ncbi.nlm.nih.gov/23962134/

37	Vinti Goel et al	2005	A proprietary extract from the echinacea plant (Echinacea purpurea) enhances systemic immune response during a common cold	Excluded because NO evidence of randomised control trail	https://pubmed.ncbi.nlm.nih.gov/16177972/
38	Carty K Y Chan et al	2020	Preventing Respiratory Tract Infections by Synbiotic Interventions: A Systematic Review and Meta-Analysis of Randomized Controlled Trials	Excluded because NO evidence of Corona virus infection	https://pubmed.ncbi.nlm.nih.gov/31996911/
39	Marina Azambuja Amaral et al	2017	Network meta-analysis of probiotics to prevent respiratory infections in children and adolescents	Excluded because NO evidence of Corona virus infection	https://pubmed.ncbi.nlm.nih.gov/28052594/
40	Lauren Fontana et al	2019	Respiratory Virus Infections of the Stem Cell Transplant Recipient and the Hematologic Malignancy Patient	Excluded because NO evidence of Corona virus infection	https://pubmed.ncbi.nlm.nih.gov/30940462/
41	Claire L Hoban et al	2019	Analysis of spontaneous adverse drug reactions to echinacea, valerian, black cohosh and ginkgo in Australia from 2000 to 2015	Excluded because NO evidence of Corona virus infection	https://pubmed.ncbi.nlm.nih.gov/31113761/

42	Shaden A M Khalifa et al	2021	Screening for natural and derived bio-active compounds in preclinical and clinical studies: One of the frontlines of fighting the coronaviruses pandemic	Excluded because NO evidence of randomised control trail	https://pubmed.ncbi.nlm.nih.gov/33067112/
43	Pius S Fasinu et al	2019	Herbal Interaction With Chemotherapeutic Drugs-A Focus on Clinically Significant Findings	Excluded because NO evidence of randomised control trail	https://pubmed.ncbi.nlm.nih.gov/31850232/
44	Sara M Handy et al	2021	HPLC-UV, Metabarcoding and Genome Skims of Botanical Dietary Supplements: A Case Study in Echinacea	Excluded because NO evidence of randomised control trail	https://pubmed.ncbi.nlm.nih.gov/33445185/
45	Kathryn M Docherty et al	2019	Soil microbial restoration strategies for promoting climate-ready prairie ecosystems	Excluded because NO evidence of Corona virus infection	https://pubmed.ncbi.nlm.nih.gov/30680826/
46	Taha Kumosani et al	2020	Evaluation in broilers of aerosolized nanoparticles vaccine encapsulating imuno-stimulant and antigens of avian influenza virus/Mycoplasma gallisepticum	Excluded because NO evidence of randomised control trail	https://pubmed.ncbi.nlm.nih.gov/32867774/

47	Grzegorz Świderski et al	2020	Spectroscopic, Theoretical and Antioxidant Study of 3d-Transition Metals (Co (II), Ni(II), Cu(II), Zn(II) Complexes with Cichoric Acid	Excluded because NO evidence of Corona virus infection	https://pubmed.ncbi.nlm.nih.gov/32664569/
48	Na Li et al	2021	Enhanced phytoremediation of PAHs and cadmium contaminated soils by a Mycobacterium	Excluded because NO evidence of Corona virus infection	https://pubmed.ncbi.nlm.nih.gov/33254925/
49	Dominique Turck et al	2020	Anxiofit-1 and reduction of subthreshold and mild anxiety: evaluation of a health claim pursuant to Article 14 of Regulation (EC) No 1924/2006	Excluded because NO evidence of Corona virus infection	https://pubmed.ncbi.nlm.nih.gov/33117457/
50	Ewa Skała et al	2020	Caffeoylquinic Acids with Potential Biological Activity from Plant In vitro Cultures as Alternative Sources of Valuable Natural Products	Excluded because NO evidence of Corona virus infection	https://pubmed.ncbi.nlm.nih.gov/32048962/
51	Andra Dumitrascu et al	2019	Acute respiratory and urinary tract infections in medical practice : a selection of complementary medicines	Excluded because NO evidence of Corona virus infection	https://pubmed.ncbi.nlm.nih.gov/31021574/
52	Maria Olga Kokornaczyk et al	2019	Phenomenological Characterization of Low-Potency Homeopathic Preparations by Means of	Excluded because NO evidence of Corona virus infection	https://pubmed.ncbi.nlm.nih.gov/30625507/

			Pattern Formation in Evaporating Droplets		
53	Shawn D Taylor et al	2019	Estimating flowering transition dates from status-based phenological observations: a test of methods	Excluded because NO evidence of Corona virus infection	https://pubmed.ncbi.nlm.nih.gov/31579602/
54	Yi Yuan et al	2021	Interventions for preventing influenza: An overview of Cochrane systematic reviews and a Bayesian network meta-analysis	Excluded because NO evidence of Corona virus infection	https://pubmed.ncbi.nlm.nih.gov/34544670/
55	Liqun Hou et al	2019	Study on the efficiency of phytoremediation of soils heavily polluted with PAHs in petroleum-contaminated sites by microorganism	Excluded because NO evidence of Corona virus infection	https://pubmed.ncbi.nlm.nih.gov/31485937/
56	Maureen L Page et al	2019	Pollinator effectiveness in a composite: a specialist bee pollinates more florets but does not move pollen farther than other visitors	Excluded because NO evidence of Corona virus infection	https://pubmed.ncbi.nlm.nih.gov/31713237/
57	Hans-Heinrich Henneicke-von Zepelin et al	2019	Non-interventional observational study broadens positive benefit-risk assessment of an immunomodulating herbal remedy in the common cold	Excluded because NO evidence of Corona virus infection	https://pubmed.ncbi.nlm.nih.gov/31074674/

58	Esin Akyüz et al	2019	Protein-Protected Gold Nanocluster-Based Biosensor for Determining the Prooxidant Activity of Natural Antioxidant Compounds	Excluded because NO evidence of Corona virus infection	https://pubmed.ncbi.nlm.nih.gov/31459484/
59	Alfredo Guarino et al	2013	Definitions and outcomes of nutritional interventions in children with respiratory infections: the approach of the COMMENT initiative	Excluded because NO evidence of Corona virus infection	https://pubmed.ncbi.nlm.nih.gov/24296796/
60	Song Mao et al	2013	Vitamin D supplementation and risk of respiratory tract infections: a meta-analysis of randomized controlled trials	Excluded because NO evidence of Corona virus infection	https://pubmed.ncbi.nlm.nih.gov/23815596/

A6. Data Extraction Template

**Source details**	Study code
	Author
	Year
	Title
**Methodology**	Study design
	Cohort
**Participant characteristics**	Sample size
	Ethnicity
	Age
	Gender
	Respiratory tract infection
	Timing of data collection
	Methodology (assessment of) for Respiratory tract pathogens (coronavirus)
	Treatment
	Screening
**Outcome (Prevalence & Epidemiology)**	Total symptom score
	Episode duration
	Area-under-curve or similar

Graphical abstract

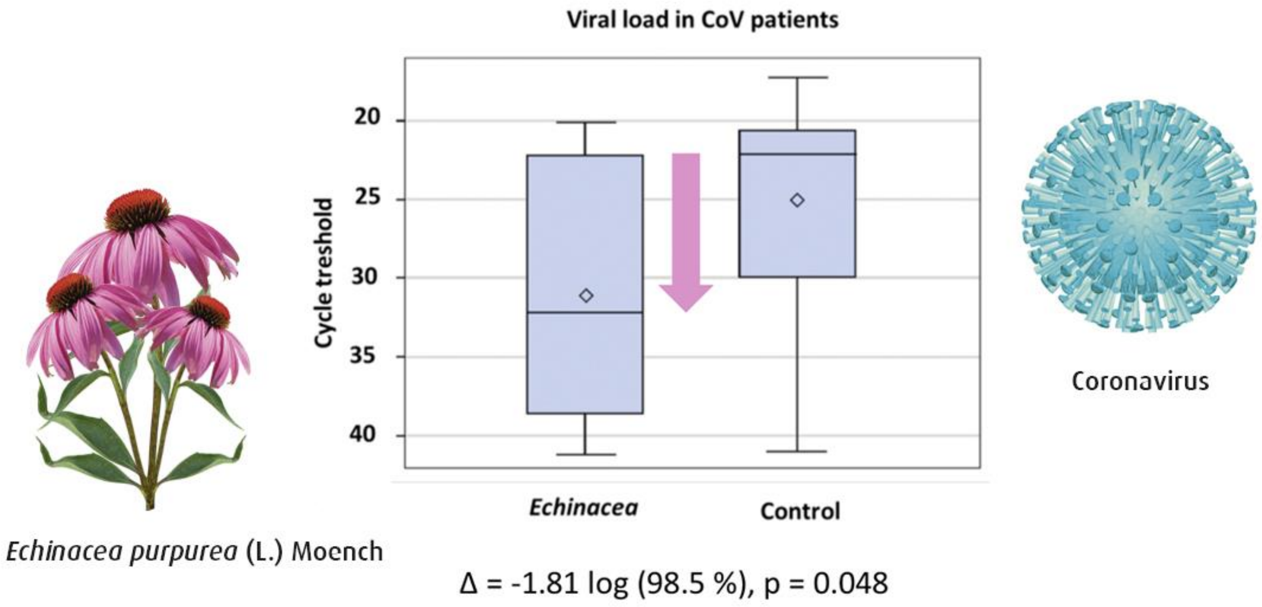

Supplement: Supplementary file 1 [file microorganisms-10-00211-s001.zip › microorganisms-1534039-SI.pdf]
